# Elastic turbulence in entangled semi-dilute DNA solutions measured with optical coherence tomography velocimetry

**DOI:** 10.1038/s41598-017-01303-4

**Published:** 2017-04-26

**Authors:** A. V. Malm, T. A. Waigh

**Affiliations:** 1 Biological Physics, School of Physics and Astronomy, University of Manchester, Oxford Rd., Manchester, M13 9PL UK; 2 Photon Science Institute, University of Manchester, Oxford Rd., Manchester, M13 9PL UK

## Abstract

The flow instabilities of solutions of high molecular weight DNA in the entangled semi-dilute concentration regime were investigated using optical coherence tomography velocimetry, a technique that provides high spatial (probe volumes of 3.4 pL) and temporal resolution (sub μs) information on the flow behaviour of complex fluids in a rheometer. The velocity profiles of the opaque DNA solutions (high and low salt) were measured as a function of the distance across the gap of a parallel plate rheometer, and their evolution over time was measured. At lower DNA concentrations and low shear rates, the velocity fluctuations were well described by Gaussian functions and the velocity gradient was uniform across the rheometer gap, which is expected for Newtonian flows. As the DNA concentration and shear rate were increased there was a stable wall slip regime followed by an evolving wall slip regime, which is finally followed by the onset of elastic turbulence. Strain localization (shear banding) is observed on the boundaries of the flows at intermediate shear rates, but decreases in the high shear elastic turbulence regime, where bulk strain localization occurs. A dynamic phase diagram for non-linear flow was created to describe the different behaviours.

## Introduction

Conventional turbulence in simple fluids, as envisaged by Osbourne Reynolds, occurs at high Reynolds numbers ($\mathrm{Re}=\frac{vL\rho }{\eta }$, where *v* is the flow velocity, *L* is the characteristic length, *ρ* is the fluid density and *η* is the fluid viscosity) in flow conditions above a threshold value (*Re* $\ge$ 5000)^1–3^. At low Reynolds numbers the non-linear terms in the Navier-Stokes equations become negligible and flows are predicted to be linear and stable.

The flow behaviors of polymeric solutions typically occur at very low Reynolds numbers (e.g. *Re* < 10^−4^ in our samples), therefore linear flows might be expected, with an absence of turbulent phenomena. However, recent progress in the analysis of complex fluids indicates that for a range of materials an analogous phenomenon of *elastic turbulence* can still occur in solutions at low Reynolds number, provided they have high levels of elasticity^4–7^. Examples of complex fluids that are thought to demonstrate this phenomenon of elastic turbulence include DNA, polyacrylamide, polyethylene oxide and worm-like micellar solutions^5, 8–14^. Elastic turbulence in complex fluids has been explored much less than conventional turbulence in simple fluids and there are large gaps in our understanding, such as the nature of the chaotic intermittency of the velocity fluctuations that are characteristic of these turbulent flows and the nature of the boundary layers. We explored the fluctuations in the velocity of picolitre volumes of opaque DNA solutions sheared in a rheometer for the first time using a newly developed method of optical coherence tomography velocimetry^9, 15, 16^. The data shows intermittency of the velocity fluctuations, a common phenomenon that is also observed in classical turbulence with simple fluids^1, 2, 5, 8^. Its’ combination with wall slip and strain localization phenomena is also explored for the first time^10, 17–21^.

The linear and non-linear rheology of DNA has been the subject of a number of previous studies with bulk rheometers^10, 18–22^. In terms of their structure and dynamics, DNA solutions can be satisfactorily modelled as flexible polyelectrolytes^23, 24^. All the DNA samples we studied were in the semi-dilute regime (overlapping chains) and are entangled (the reptation model can be used to explain their dynamics)^25^.

A major barrier to studies of flow phenomena is the paucity of high resolution techniques to study high concentration opaque specimens in combination with their viscoelasticity^26^. The velocimetry of concentrated DNA solutions has thus been little studied in the literature.

Optical coherence tomography (OCT) is a newly developed imaging technique (1991) that uses the finite coherence length of a light source to act as a coherence gate to reduce the effects of multiple scattering^27, 28^, and it can probe up to ~2 mm thicknesses of human tissue or opaque complex fluids using infrared light. Doppler shift OCT is an extension on this methodology that uses the spectroscopic detection of the energies of quasi-elastically scattered infrared photons to calculate the absolute velocity of sheared complex fluids^29–33^. Our Doppler shift OCT apparatus has been optimised to measure the velocity of 3.4 pL volumes of complex fluid at different positions inside the gap of a stress-controlled rheometer. It performs well at much higher shear rates than conventional Doppler OCT devices due to its time domain nature (most commercial designs are now frequency domain), high data acquisition rates and the use of a power spectral analysis for the fringe signal, which is more robust than direct phase retrieval methods^16^. An extension over our previous OCT rheometer design is that we now use software demodulation of the Doppler shifted signal, which allows more convenient quantification of the velocity fluctuations experienced by complex fluids under flow, which is extremely useful for characterizing unstable shear flows^16^.

Both shear banding and wall slip have been observed previously in high elasticity DNA solutions when they are sheared at reasonably high rates^10, 17, 19^. However, the shear banding was found to be a transient phenomenon and it was not observed in fully developed flows, unlike wall slip which was a steady state phenomenon in the solutions. Electrostatic interactions between DNA chains are also thought to play an important role in flow instabilities, with wall slip increasing at high salt concentrations^34^. Interestingly in the current study we find evidence for strain localization (equivalent to shear banding^35^) on the walls of the boundaries, which has been previously suggested by Steinberg *et al*. to be the source of the elastic stresses that drive elastic turbulence^36^. Bulk strain localization (shear banding) is observed in fully developed elastic turbulence in our study, which was performed at much higher shear rates than those previously studied^19^.

The original study that coined the terminology *elastic turbulence* with polyacrylamides purported to be in the dilute concentration regime, just below the semi-dilute overlap concentration^5^. However, the overlap concentration is notoriously difficult to calculate with polyelectrolytes and our own microrheology measurements indicate that the polyacrylamides studied may well have been in the semi-dilute regime^37^, in agreement with the results of the Larson group^7^. A similar ambiguity is observed in some of the literature on DNA elastic turbulence and the studies have again been performed at the boundary of the semi-dilute overlap regime^38^ (SI Section 7). In the current study we examine elastic turbulence in concentrated solutions, which are well above the semi-dilute overlap concentration and the solutions are also entangled i.e. reptating. Analogous elastic turbulence phenomena have previously been observed in entangled semi-dilute worm-like micelles^12^, polyethylene oxides^39^ and polyacrylamides^40^. There is evidence for a separate elastic inertial turbulence regime in dilute polymer solutions at very high shear rates, although it is thought to be a separate phenomenon from conventional elastic turbulence^6, 41^.

DNA served as a standard monodisperse example of a flexible polyelectrolyte in our studies and the elastic turbulence phenomena observed are expected to be generic over a wide range of solution state polymers. Studies of DNA viscoelasticity also have applications in biology, including the treatment of cystic fibrosis and microbial infections in lungs, where large amounts of extracellular DNA in biofilms often occur. Extracellular DNA is also now thought to provide an important contribution to the viscoelasticity of the mucus layer found on the walls of intestines, which may affect food absorption and microbial infections^42^. Many techniques in genetic engineering involve the microfluidics of DNA chains. High throughput devices often require high DNA concentrations and elastic turbulence phenomena are also known to occur in microfluidic flows^43–45^.

The boundary layer for polymeric elastic turbulence has been subject to a few previous studies, that used a curved microfluidic channel to investigate the flow profile using particle imaging velocimetry in transparent low concentration samples^46–48^. The previous microfluidic studies stressed the difference between the spatial dependence of the velocity fluctuations in elastic turbulence compared with that observed in classical isotropic turbulence (CIT). The spatial variation of velocity fluctuations is relatively smooth with elastic turbulence (opposite to CIT), whereas the temporal fluctuations are very large (similar to CIT), but with a different non-Kolmogorov power spectrum of the fluid velocities (*P*(*ω*) *~* *ω* ^*−α*^, *α* = *5/3* for classical isotropic turbulence predicted by Kolmogorov using the Taylor frozen turbulence hypothesis^49^, whereas *α* > 3 for elastic turbulence).

Historically, polymer additives at low concentrations have had a wide range of technological applications in turbulent drag reduction and consequently a large amount of research has been performed on them. However, here we focus on the relatively unexplored regime of extremely high polymer concentrations where solution elasticity becomes more important^50^. Numerous applications of these elastic turbulence phenomena have recently been found in microfluidics, such as turbulent mixing^43, 47^. Elastic turbulence has a big impact on the energy dissipated in industrial flows of high concentration polymers modifying their processing conditions^5^ and it may be related to other non-linear instabilities, such as the shark-skin effect that is important in extrusional flows^51^.

## Results

**Figure 1:**
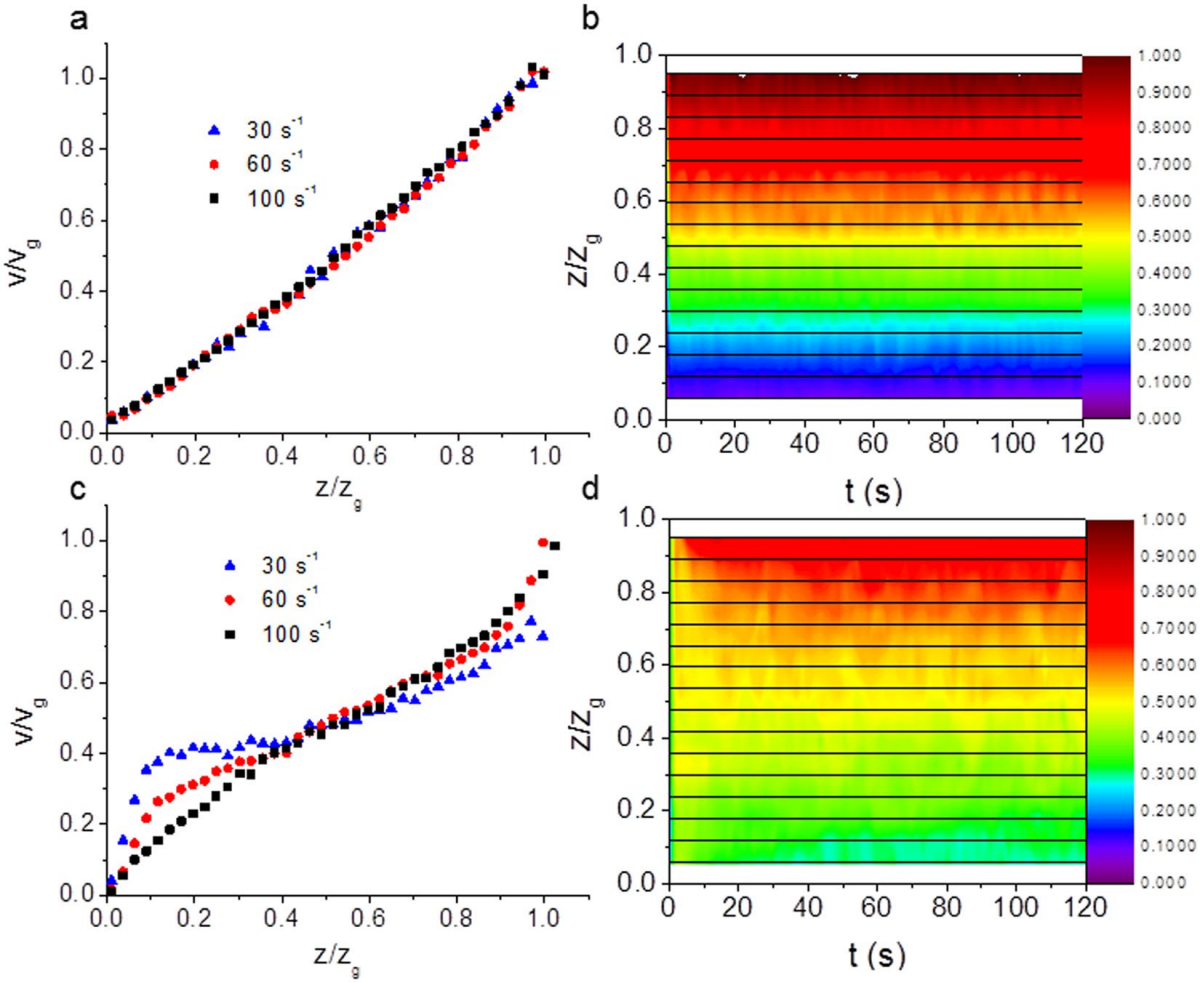
(**a**) Normalized shear velocity (*v*/*v*_*g*_) as a function of normalized distance across the rheometer gap (*z*/*z*_*g*_) for a 5 mg/ml solution of DNA (high salt) at three different steady shear rates which show linear Newtonian-like behavior i.e. a constant velocity gradient. (**b**) A colour map of shear normalized velocity of the same solution as a function of the normalized distance across the rheometer gap and the time after shear start-up at a constant shear rate of 60 s^−1^. The velocity profiles demonstrate a stable steady state. The horizontal lines show the depths at which measurements were made and the colour scale is normalized to the top plate velocity. Similar data for 10 mg/ml solutions of DNA (high salt) are shown in (**c**) and (**d**), with all of the velocity profiles showing significant wall slip. Some strain localization at the boundaries is also seen at all shear rates in (**c**). Using the colour map scale, wall slip is identified by the wide yellow/green region, which is particularly clear when (**d**) is compared to (**b**).

5 mg/ml DNA solutions display linear behaviour for the velocity as a function of gap position with only a slight degree of wall slip at the upper and lower plates, Fig. 1a. The velocity colour map as a function of time for the 5 mg/mL DNA solutions is shown in Fig. 1b. Repeat measurements of the velocity profiles for the 5 mg/ml concentration solutions were found to be reproducible, with the velocity remaining stable over time, as seen in the colour map (Fig. 1b). Velocity profiles were also measured for shear rates up to 200 s^−1^ and these also showed linear flow behaviour with no apparent turbulence.

**Figure 2:**
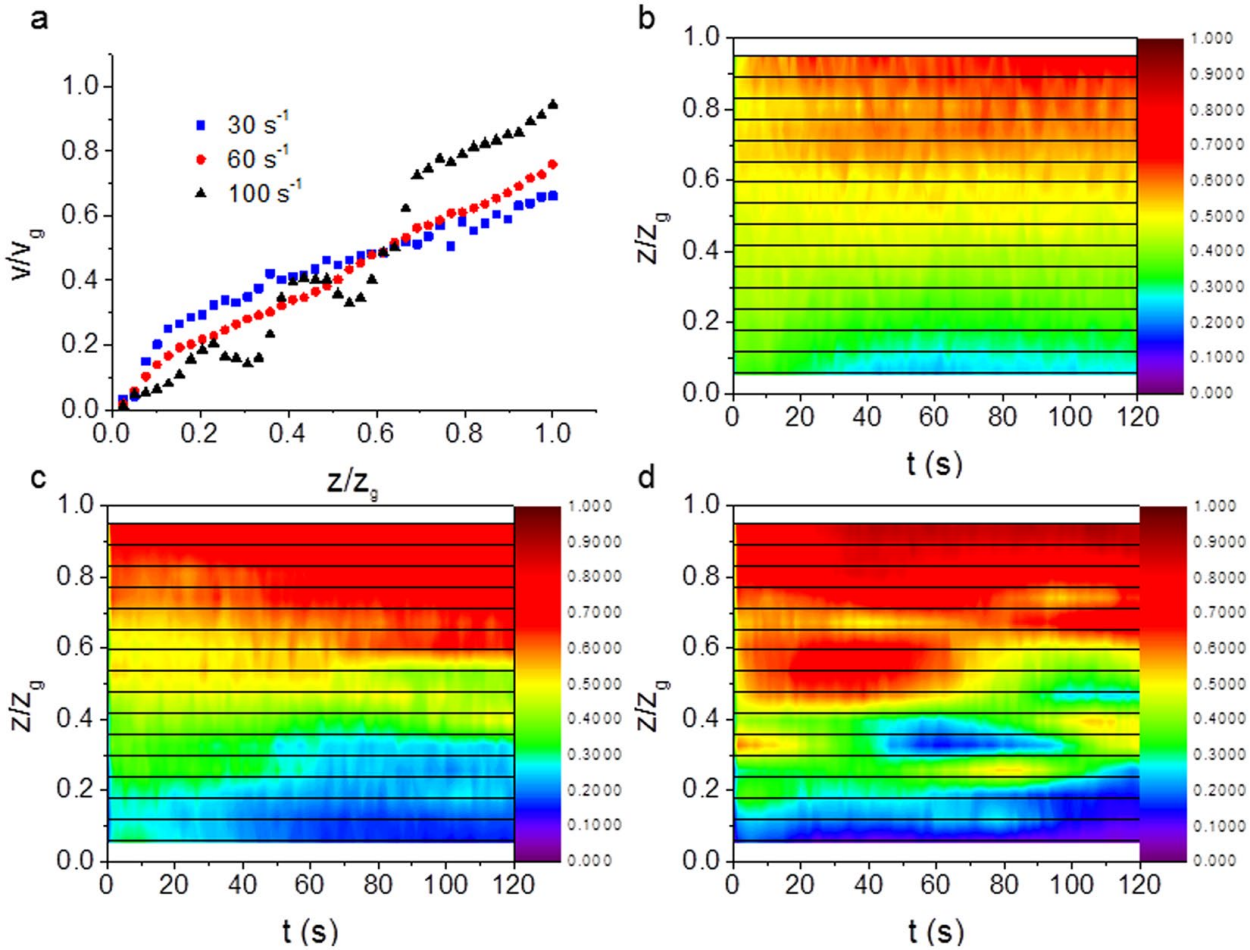
(**a**) Normalized shear velocity (*v*/*v*_*g*_) as a function of normalized distance across the rheometer gap (*z*/*z*_*g*_) for a 15 mg/ml solution of DNA (high salt) at three different steady shear rates that show significant wall slip, bulk strain localization and irregular flow when sheared at 100 s^−1^. Strain localization is also observed on the boundaries, but it decreases with shear rate. The colour maps show the normalized shear velocity as a function of the normalized distance across the rheometer gap, for the same sample and the time after shear start-up (*τ*), at constant shear rates of (**b**) 50 s^−1^, (**c**) 60 s^−1^ and (**d**) 100 s^−1^. The horizontal lines show the depths at which measurements were made, and the colour scale is normalized to the top plate velocity.

For higher DNA concentrations, at 10 mg/mL, significant wall slip was observed and the greatest shear gradients occurred near the plates (Fig. 1c). A greater degree of wall slip occurred at lower shear rates. The velocity profiles were again reproducible and the colour map in Fig. 1d shows that the flow is relatively stable away from the wall boundaries. Strain localization is observed at both the boundaries i.e. curvature in the velocity gradient. As the DNA concentration is increased to 15 mg/mL, wall slip behaviour continues to be observed with some strain localization on the boundaries, however irregularities in the time averaged velocity profiles are also observed above a critical shear rate, (Fig. 2a) i.e. bulk strain localisation occurs.

The flow for the 15 mg/ml DNA solutions becomes unstable at shear rates greater than 60 s^−1^ and irregular velocity profiles are measured for all applied shear rates above this critical value. Whilst the ensemble averaged statistics of the irregularities are robust for repeated measurements (Fig. 2a), the velocity profiles themselves as a function of time are highly variable (Fig. 2d). Specifically at 100 s^−1^ the DNA solutions show robust repeatable oscillatory profiles of velocity as a function of position in the gap when averaged over time (Fig. 2a), which is associated with bulk strain localization (shear banding). The colour map figures (Fig. 2b,c,d) show that the flows with shear rates ≤60 s^−1^, always show wall slip, which evolves over time in the range 30–60 s^−1^, whereas at higher shear rates, intermittent instabilities are observed, where the magnitude of the shear velocity can change abruptly.

**Figure 3:**
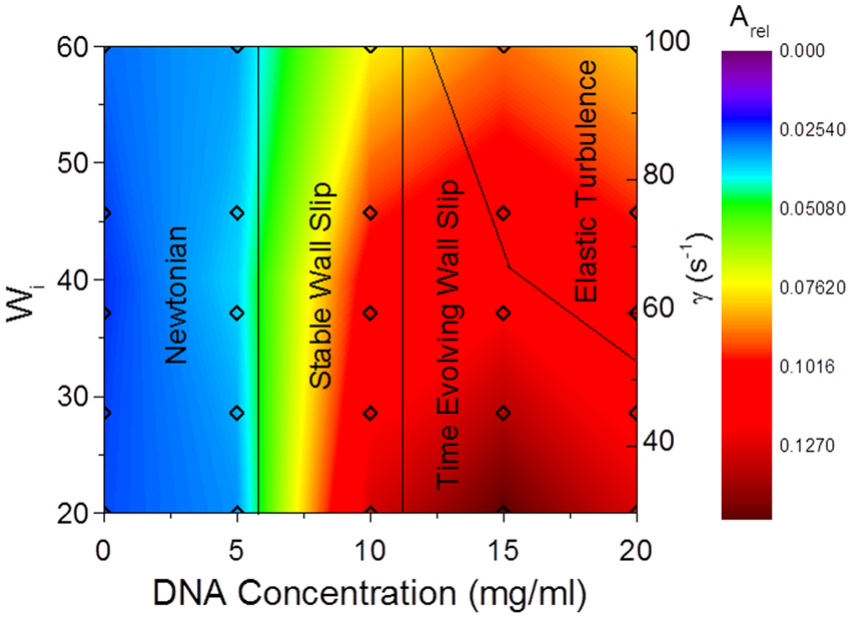
Phase diagram of the different types of flow for high salt DNA solutions as a function of the Weissenberg number (proportional to the applied shear rate ($\dot{\gamma }$) and the concentration of the DNA). The colour map shows values of the integrated area of each velocity profile as a function of depth, subtracted from the constant gradient expected for Newtonian-like flows (*A*_*rel*_), measured at the points indicated by hollow diamonds. The boundaries between the different flow regimes are also shown. The shear rates have been rescaled to give the Weissenberg number (*W*_*I*_) to facilitate comparison between experiments.

A dynamic phase diagram was constructed based on the results (Fig. 3).

### Velocity fluctuations in elastic turbulence

**Figure 4:**
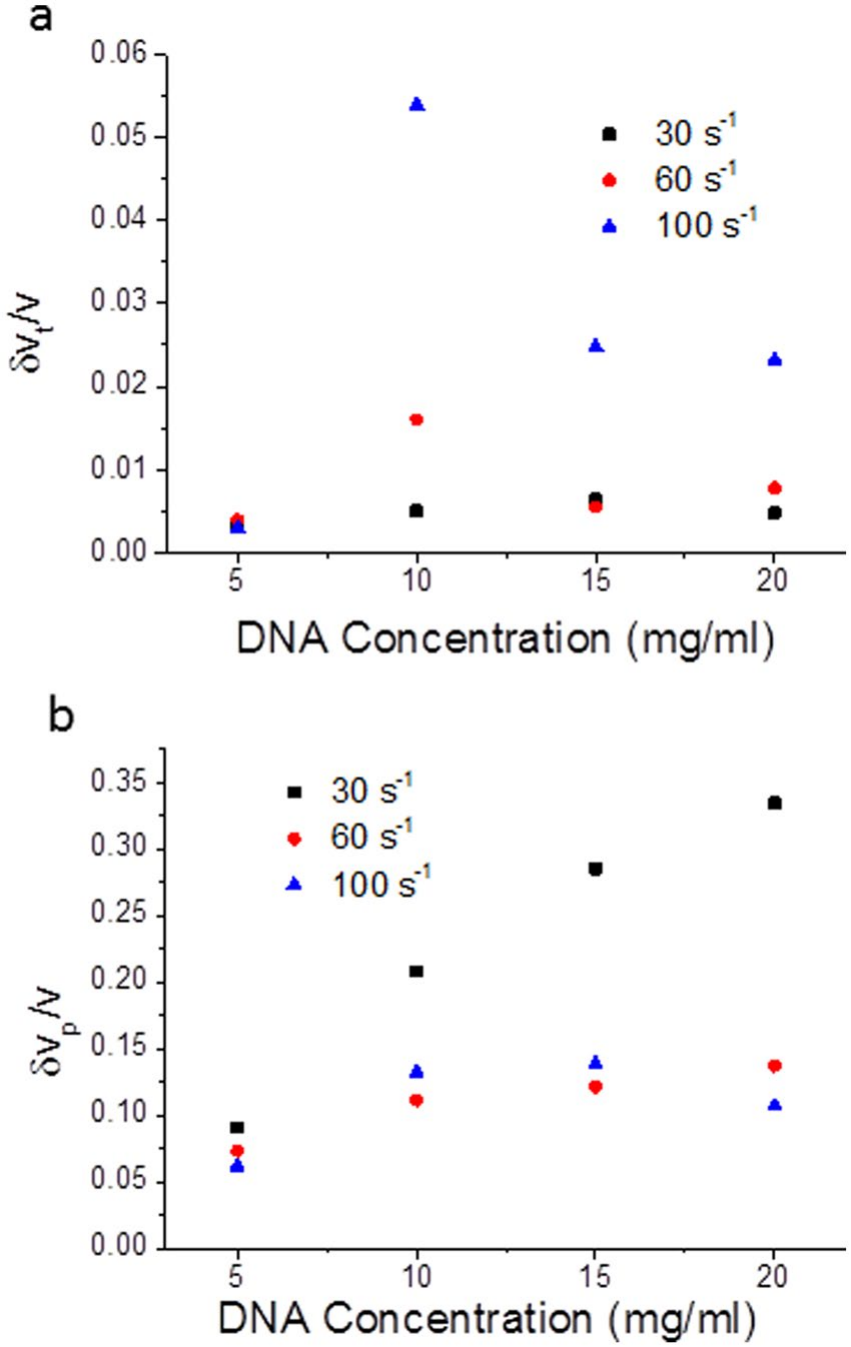
Reduced velocity fluctuations (*δv/v*) averaged across the whole gap as a function of DNA concentration (high salt) for a range of applied shear rates calculated from, (**a**) the time variation of the measured velocity (long times, 1 s), and (**b**) the width of the measured Doppler signal peak (short times, 40 ms).

The velocity profiles as a function of gap position (Figs 1 and 2) are very useful for understanding the steady state behavior of the flows. However, they provide little information about instabilities in the flow characteristics of elastic turbulence. Fortunately, the velocity fluctuations could also be measured, which increase with shear rate in long time measurements (Fig. 4a). However, at short times and high DNA concentrations the order reverses with high amplitude velocity fluctuations occurring at lower shear rates (Fig. 4b).

**Figure 5:**
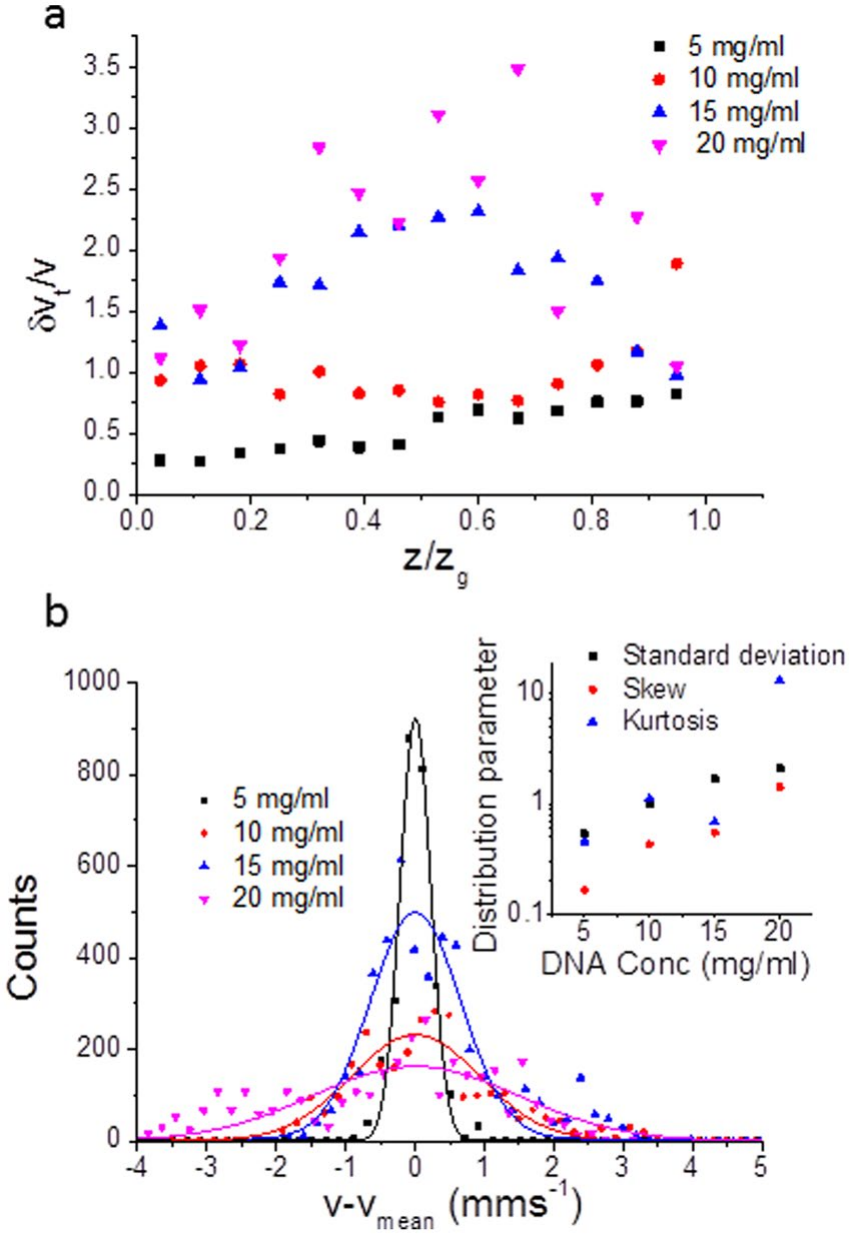
(**a**) Reduced velocity fluctuations (*δv*/*v*) as a function of normalized distance (*z*/*z*_*g*_) across the rheometer gap (500 μm) for different concentrations of high salt DNA solution sheared at 60 s^−1^, measured for long time distributions of the velocity. (**b**) Histograms (proportional to the probability distribution function) of long time (1 s) velocities, measured close to the lower plate of the rheometer, for solutions with a range of DNA concentrations at high salt with an applied shear rate of 60 s^−1^. Gaussian fits are plotted for each data set to aid the eye, although the probability functions are only well described by Gaussians at low DNA concentrations. Fat-tailed distributions occur at high DNA concentrations. The velocities (*v*) are plotted as a function of their distance from the mean velocity (*v*_*mean*_) to allow for the slight effects of wall slip. The inset shows the absolute values of characteristic statistical parameters of the velocity distribution as a function of DNA concentration averaged over the entire rheometer gap i.e. standard deviations (black squares), skewes (red circles) and kurtosises (blue triangles).

Measurement of the long time velocity fluctuations as a function of normalized depth across the rheometer gap can assist in identifying regions of different flow. The amplitude of the velocity fluctuations (*δv*_*I*_ */v*) was measured as a function of depth in the sample (Fig. 5a). At low DNA concentrations the velocity fluctuation amplitudes are fairly constant as a function of depth, whereas at higher DNA concentrations the amplitudes are lower near to the plates. By histogramming the measured velocity over long times, the distribution of velocities can also be analyzed, Fig. 5b. In agreement with earlier observations of the velocity fluctuations, the probability distribution of measured velocities is narrow for the 5 mg/ml DNA concentration solution, but as the concentration was increased, the distributions become broader with skewed tails (Fig. 5b). To avoid making assumptions as to the form of the distributions of the measured velocity, fits were not used as a means of quantification; instead the standard deviation, skew and kurtosis were calculated and plotted for comparison in Fig. 5b inset. All three of these parameters increase with DNA concentration and a dramatic increase is seen for the kurtosis in the elastic turbulence regime.

As discussed previously, the velocity fluctuations at long times are significantly higher for 15 mg/ml DNA concentration solutions which displayed turbulence. However, the velocity fluctuations measured are seen to decrease towards the rheometer plates (Fig. 5a), suggesting the presence of a boundary layer which contains some strain localization (Fig. 2a).

**Figure 6:**
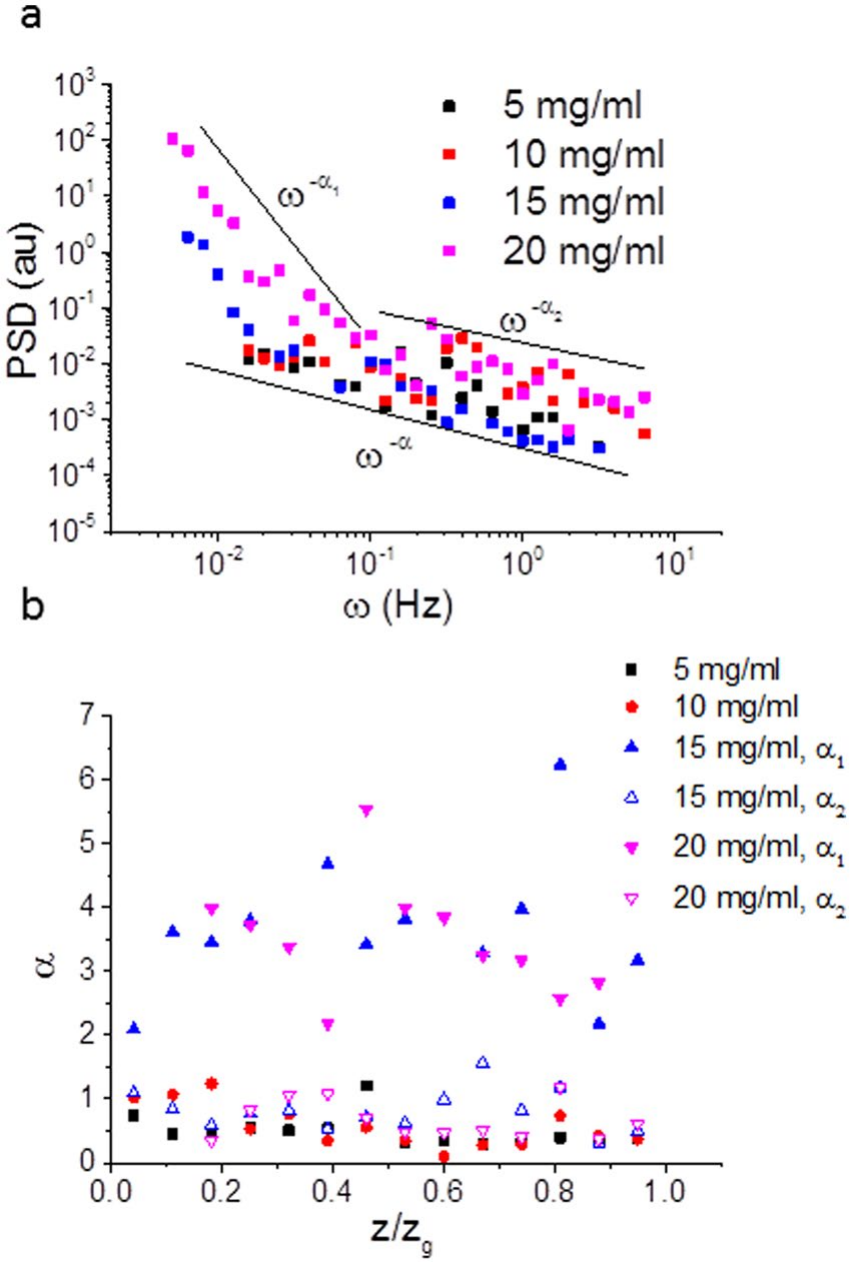
(**a**) Power spectral density (PSD) as a function of frequency (*ω*) of the velocity fluctuations measured for four different concentrations of high salt DNA solution (5, 10, 15, 20 mg/mL), all with an applied shear rate of 60 s^−1^. (**b**) Values of the power law exponent (*α*) of the power spectral density (*P*(*ω*) *~* *ω* ^*−α*^) as a function of normalised distance (*z*/*z*_*g*_) across the rheometer gap. Open (no turbulence) and closed (turbulence) symbols are used to distinguish between the two observed power law dependencies when turbulence occurs.

The power spectral density (*P*(*ω*), *ω* is the frequency) of the velocity fluctuations over time shows a distinct power law dependence, with *P*(*ω*) *~* *ω* ^*−*0.5+/−0.1^ for the 5 and 10 mg/ml DNA concentrations (Fig. 6a). At higher concentrations where elastic turbulence is observed, there are two separate power law dependencies. At high frequencies (>0.1 Hz), the power law is similar to that of the non-turbulent viscoelastic flows at lower concentrations, however at low frequencies (<0.1 Hz), a much higher value for the power law exponent is measured with *P*(*ω*) *~* *ω* ^*−*3.5+/−0.8^, Fig. 6a. For comparison, with dilute silica bead tracers in water at high Reynolds number, with no added DNA (supplementary information, Fig. S5) the exponents are between those at low and high DNA concentrations, *P*(*ω*) *~* *ω* ^*−*1.1+/−0.1^.

Plotting the power law dependence of the power spectral density as a function of normalised distance across the rheometer gap (Fig. 6b) shows no clear differences near to the plate boundaries. In the turbulent regime the boundary layer has intermittent velocities and is also turbulent, although the amplitude of the fluctuations are reduced near to the boundaries (Fig. 5a).

### Low salt concentration elastic turbulence in DNA

DNA is a highly charged polymer and reduction in the amount of salt in their solutions is known to cause a large increase in chain size due to increased electrostatic repulsion, which can increase the viscosity and elasticity of the solutions^52^. Repulsion of the DNA chains from the plates of the rheometer would also be expected to increase in low salt solutions, due to electrostatic repulsion decreasing adhesion, which consequently could affect both wall slip and strain localization behaviour.

**Figure 7:**
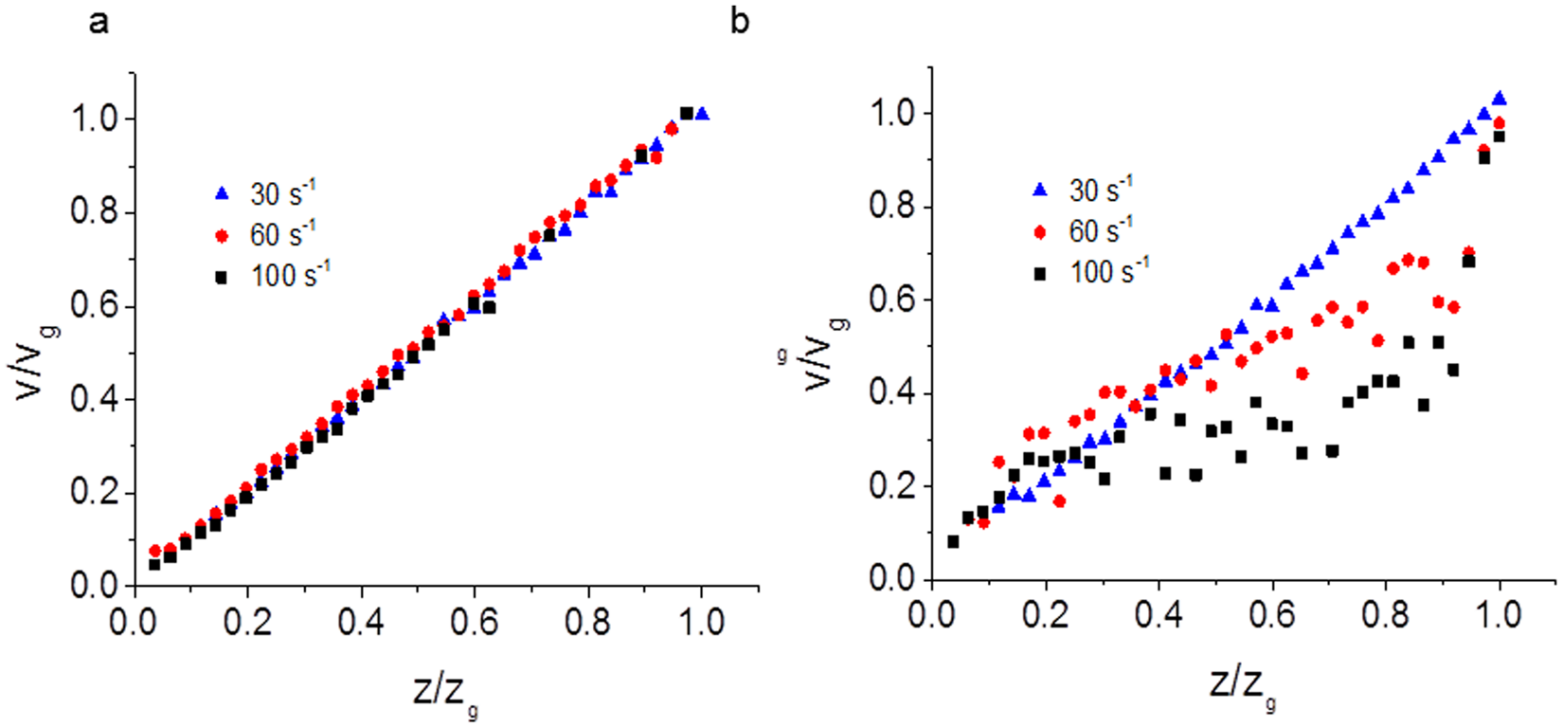
Normalized shear velocity (*v*/*v*_*g*_) as a function of normalized distance across the rheometer gap (*z*/*z*_*g*_) at three different steady shear rates for (**a**) 10 mg/ml DNA solutions in water (low salt), that show linear Newtonian-like behavior, and (**b**) 15 mg/ml DNA solutions in water (low salt), that show a transition between linear Newtonian-like behavior at low shear rates and wall slip at higher shear rates.

**Figure 8:**
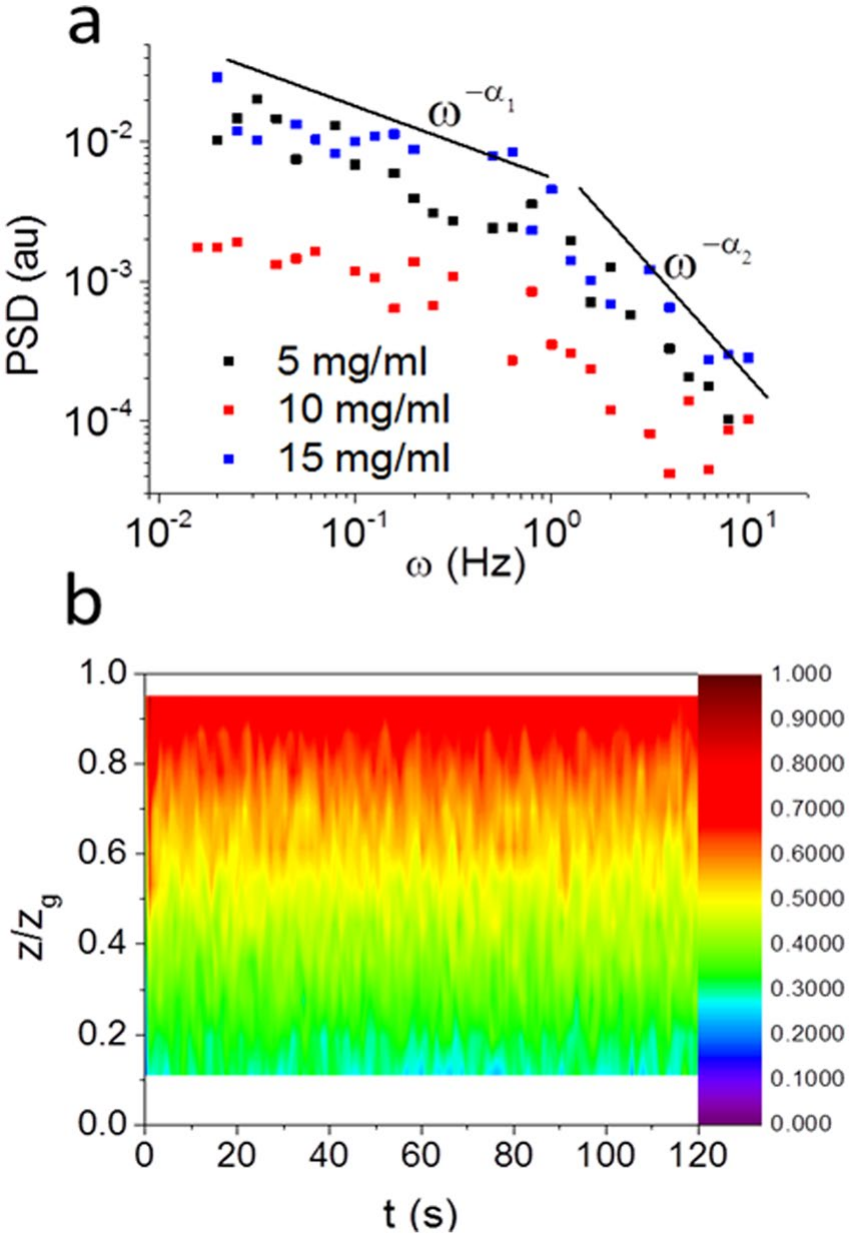
(**a**) Power spectral density (PSD) of the velocity fluctuations measured for three different concentrations of low salt DNA solutions, all with an applied shear rate of 60 s^−1^. Two separate power law behaviors are observed, at low frequencies $P(\omega )\sim {\omega }^{-{\alpha }_{1}}$ and at high frequencies $P(\omega )\sim {\omega }^{-{\alpha }_{2}}$. (**b**) Colour map of shear normalized velocity as a function of the normalized distance across the rheometer gap and time after shear start up for a constant shear rate of 60 s^−1^. Intermittent fluctuations in the velocity are observed, although at much shorter times than in Fig. 2d.

Reduction of salt in the DNA solutions (physiological salt reduced to no added salt conditions) in the velocimetry experiments reduced the amount of wall slip observed (compare Fig. 7 with Fig. 1). At high DNA concentrations and high shear rates the low salt DNA solutions also demonstrated intermittent velocity fluctuations (Fig. 8b). The power spectral density also had high values of the exponents for the power law decay ($P(\omega )\sim {\omega }^{-\alpha }$, *α* > 3). However, the power law decay of the PSD occurred at substantially higher frequencies (two orders of magnitude higher frequencies) than those of high salt solutions (compare Fig. 8a and Fig. 6a).

## Discussion

### Dynamic phase diagram for wall-slip and elastic turbulence

All the DNA solutions we examined were above both the semi-dilute concentration and the entanglement concentration of the chains, so they are expected to be overlapping and reptating^25^. All the DNA solutions examined shear thinned in the non-linear flows used (supplementary information Fig. S1a), which is typical of DNA and polyelectrolyte solutions in general^21^. A stress overshoot is also observed in these non-linear flows (Fig. S1b), which is common in entangled long flexible polymers and is thought to be related to the relatively long relaxation times of these materials i.e. the chains require times on the order of a few seconds in order to react to the sudden application of stresses (determined by the characteristic reptation time of the chains)^20^.

As the DNA concentration was varied in high salt conditions the dynamic phase diagram (Fig. 3) has a series of regimes. At low DNA concentrations (5 mg/mL) the solutions are Newtonian-like (there is a constant velocity gradient across the gap, although the samples still shear thin in bulk rheology experiments) and there are no substantial fluctuations in the fluid velocities as a function of time (Fig. 1a,b). As the DNA concentration is increased (10 mg/mL), wall slip occurs, and the slip length increases with shear rate (Fig. 1c,d). At DNA concentrations of 15 mg/mL, at low shear rates a wall slip regime is again observed, but the slip length evolves with time (Fig. 2a,c). We conclude the longer relaxation times of the DNA chains at higher concentrations (on the order of many seconds), gives rise to the stress overshoot in rheology measurements (Fig. S1) and cause the slow evolution of the wall slip towards a steady state. Wall slip is affected by adhesion, lubrication and hydrodynamics effects at the surface (e.g. syneresis of the solvent out of the concentrated solutions)^53, 54^. There is expected to be higher wall slip with the longer Debye screening lengths at low salt concentrations due to stronger electrostatic repulsion and thus improved lubrication (opposite to the effect observed experimentally), but slip is also affected by both the viscosity and elasticity of the bulk fluid, which are both increased at low salt concentrations.

At higher shear rates a robust reproducible steady state profile is observed for the average velocity as a function of position, which has an oscillatory profile and a limited amount of wall slip is observed (Fig. 2a), whereas the velocity as a function of time (Fig. 2d) shows that intermittent chaotic fluctuations occur at short times typical of elastic turbulence. The boundary between the time evolving wall slip and elastic turbulence depends on the Weissenberg number (Fig. 3, with a curved boundary) i.e. there is a critical shear rate (or equivalently Weissenberg number) for the onset of elastic turbulence as observed previously with low concentration polyacrylamides^5^.

Strain localization at the boundaries is observed at 10 mg/mL DNA with shear rates >30 s^−1^ (Fig. 1c). Strain localization moves into the bulk at high shear rates (>100 s^−1^) and high DNA concentrations (≥15 mg/mL, Fig. 2a), but it is accompanied by intermittency in the velocity profiles and thus elastic turbulence. These shear rates are much higher than previously used to explore shear banding in DNA samples^19^.

### Velocity fluctuations in elastic turbulence

The velocity fluctuations were measured at both short and long times (Fig. 4). Interestingly the fluctuations at high shear rates have higher amplitudes at long times (Fig. 4a), whereas the order reverses at short times and high DNA concentrations (Fig. 4b). Currently we have no clear explanation of this phenomenon.

The standard deviation of the velocity distributions is shown in Fig. 5a as a function of position across the gap. For the elastic turbulent specimens (15 and 20 mg/mL) regions of reduced amplitude velocity fluctuations were observed at both the upper and lower plates. These regions of reduced amplitude velocity fluctuations may be related to an elastic turbulent boundary region that has been observed previously in microfluidic flows of low concentration polyacrylamides^46–48^. The boundary regions are thus relatively large, on the order of 100 μm (20% of the rheometer gap size, 500 μm) on either of the plates.

The probability distribution functions of the velocities at long times (Fig. 5b) and high shear rates (60 s^−1^), time averaged at a point close to the lower plate, demonstrate a clear evolution in their behavior as a function of polymer concentration. At low polymer concentrations the velocity fluctuations are narrowly distributed Gaussian functions as expected in Newtonian-like flows. However, for high polymer concentrations the velocity fluctuations broaden considerably and become non-Gaussian with fat-tails superposed on the distributions. Such fat-tails are characteristic of the intermittent phenomena observed in classical turbulence^3^.

Figure 5b inset demonstrates a number of non-Gaussian features for the probability density function of the velocity fluctuations. Clearly the kurtosis (a measure of how non-Gaussian a function is), averaged over the entire gap, increases dramatically with polymer concentration as the velocity distributions demonstrate their fat-tails during elastic turbulence. Similarly, there are dramatic increases in the magnitude of the fluctuations (the standard deviation) at high DNA concentrations during elastic turbulence and the distributions demonstrate an increased degree of asymmetry (the skewness increases).

There is a single analytic theory in the literature that describes the velocity fluctuation spectra of turbulent polymeric fluids^55–57^. The theory is successful in that it predicts a sharp transition in the frequency dependence of the power law fit to the power spectral densities (PSD) from low to high exponents (*α* > 3) during the onset of elastic turbulence (Fig. 6b) which is observed in our data. However, the theory is derived for dilute polymeric solutions, which calls into question its applicability with the concentrated DNA solutions that we examined. The sharp switch of the PSD exponents of the velocity fluctuations we observed as a function of polymer concentration, from less than one to greater than three (Fig. 6), are in broad agreement with that observed previously in a particle imaging velocimetry study with polyethylene oxide solutions as a function of polymer concentration^39^. However, PEO is known to gel at high concentration, unlike DNA, which potentially complicates these previous studies. A similar switch from low to high exponents of the PSDs as a function of concentration was also observed for worm-like micelles^13^.

### Low salt concentration elastic turbulence in DNA

The reduction of salt in the DNA solutions (from a physiologically relevant buffer to no added salt conditions) shifts the appearance of the wall slip regime to higher DNA concentrations. Thus there is no wall slip observed in 10 mg/mL low salt solutions (Fig. 7a) in contrast to that seen with high salt solutions (Fig. 1c). At 15 mg/mL, low salt DNA solutions again show wall slip (Fig. 7b). Low salt solution DNA samples have slightly larger viscosities and elasticities than their high salt equivalents (SI, Fig. S3), which reflects the effects of charge screening in reducing the size of polymer chains (smaller, high salt, charge-screened polyelectrolyte chains provide lower viscosities and fewer entanglements). In terms of dynamic scaling theories, the chains are expected to switch from being flexible polyelectrolytes to being flexible neutral polymers as the salt is increased^52^.

Wall slip might be expected to increase in low salt concentrations due to increased electrostatic repulsion (opposite to the behaviour observed), but it appears to be counteracted by the increased elasticity and viscosity of the bulk solutions that tend to decrease the size of the boundary layer^10^. Some strain localization still occurs on the boundaries at higher shear rates >30 s^−1^ (Fig. 7b).

Interestingly, the low salt conditions also have a dramatic effect on the velocity fluctuations in DNA solutions (Fig. 8a). The sharp power law decrease of the PSD (*PSD* ~ *ω* ^*−α*^, with *α* > *3*) shifts by 2 orders of magnitude to higher frequencies and the data now has a plateau at lower frequencies. This low frequency plateau in the PSD has been observed previously with polyacrylamides, which can also act like flexible polyelectrolytes^5, 37^. The change in the short time intermittency with a reduction in salt is also clear in the velocity/time plots (compare Fig. 8b with Fig. 2d).

In the supplementary information section (Fig. S6) plots of the power spectral density of the velocity are shown for solutions with no DNA, at a high Reynolds number (*P*(*ω*) ~ *ω* ^*−1*.*1*+*0*.*1*^). These results are reasonably close to the Kolmogorov prediction of α = −5/3 for classical isotropic turbulence.

### General phenomenology

Clearly there are a wide range of open questions in our understanding of elastic turbulence in DNA solutions, but we hope to have demonstrated that optical coherence tomography provides a high resolution probe for a range of novel non-linear flow phenomena. In the future it would be useful to measure the velocity fluctuations as a function of the chains’ molecular weight to accurately determine the critical thresholds on both the solutions’ Weissenberg numbers and the elasticities that are needed for elastic turbulence and wall slip to occur. However, the current measurements do provide a useful first step in demonstrating the existence of elastic turbulence, wall slip and shear banding in concentrated DNA solutions.

The standard theoretical model for elastic turbulence provided by Steinberg *et al*. is that the chaotic motion is spatially smooth, but random in time^36, 47, 58–60^. The high value of the velocity PSD decay exponent is the result of stretching and folding of the elastic stress field, which transfers energy from small to large length scales, the direct opposite of the explanation of intermittency in conventional turbulence^2, 3^. In conventional high Reynolds number turbulence intermittency is due the generation and breaking of eddies (large to small length scales), whereas in elastic turbulence elastic stresses are injected from the boundary layers into the bulk flow. A new perspective is given in this study by consideration of strain localization, which is a standard non-linear phenomenon in elastic solids and would be expected to be a limiting behavior at very high solution concentrations^35^. Strain localization at the boundaries may provide the necessary elastic stresses to drive the elastic turbulence in the flows. The amount of boundary strain localization is reduced in our studies after the onset of fully developed elastic turbulence, which results in bulk strain localization (shear banding).

Practically, wall-slip, boundary confined strain localization (shear banding on the walls) and elastic turbulence have a number of important implications for polymer processing. The power injected into a flow, that is required to transport molecules, increases substantially in the elastic turbulent regime and it is known to closely follow the spectra of the velocity fluctuations^58^. Thus pumping of microfluidic flows is considerably easier in the Newtonian-like constant shear gradient regime (energetically), but the extra costs in the elastic turbulence regime may be warranted if improved mixing is required. A problematic phenomenon in polymer processing is the shark skin effect in extruded materials and it is expected that this phenomenon is closely related to elastic turbulence i.e. it can be avoided outside the elastic turbulence regime. Elastic turbulence also has applications in oil recovery^40^ and food processing^61, 62^.

Elastic turbulence could be explored in a wide range of other industrially important materials, such as opaque polymer melts and soft solids (tomato ketchup, margarine etc. ref. 15) with OCT velocimetry. Bacterial turbulence (internally driven by the bacteria’s motors) also occurs at low Reynolds numbers and could also be measured using the device^63^. Inclusion of a second fiber in the OCT design would also allow the full *structure function* of elastic polymeric turbulence to be measured, i.e. the full spatial dependence of the velocity fluctuations^1, 2^, which is neglected in the current study.

OCT velocimetry has thus been successfully used to explore the non-linear flow behavior of high concentration DNA solutions for the first time. In high salt solutions, as the DNA concentration is increased, the flow switches between Newtonian-like, wall slip and evolving wall slip regimes. At high shear rates and high DNA concentrations, elastic turbulence occurs, which is accompanied by fat-tailed distributions of the intermittent velocities, measured in a 3.4 pL volume, and a characteristic power spectral density of the velocity fluctuations, $P(\omega )\sim {\omega }^{-3.5\pm 0.8}$. In low salt solutions wall slip is greatly reduced. Elastic turbulence is also observed in low salt DNA solutions, but the characteristic power law decrease of the power spectral density of the velocity fluctuations shifts to much higher frequencies. A considerable boundary layer is tentatively observed in high salt elastic turbulence (20% of the gap size), but the spectra of its velocity fluctuations are very similar to those of the bulk fluid, although the amplitude of its variance decreases at the boundaries. Strain localization (shear banding) on the boundaries is observed at intermediate shear rates and intermediate DNA concentrations. Bulk strain localization (shear banding) is accompanied by velocity intermittency in fully developed elastic turbulent flows.

## Methods

OCT velocimetry can be classified as a new form of non-linear microrheology^64, 65^. It compares favourably with a number of other methods to probe non-linear flows such as MRI, acoustic imaging, confocal microscopy, laser Doppler velocimetry and particle imaging velocimetry methods^26^ (SI Sections 2, 8).

### Optical Coherence Tomography Apparatus

A stress controlled rheometer (Bohlin Gemini) with a transparent plate-plate geometry was used to perform shear ramp and steady shear rate experiments with a plate separation of 500 μm. The rheometer was connected to a fiber based interferometer for OCT velocimetry measurements, as described previously^16^. The interferometer allowed the velocity of the fluids to be measured at specific depths within the sample volume. Measurements were made on 9 μm thick slices of sample with a probe volume of 3.4 pL. The technique is capable of measuring a wide range of time scales and the use of infrared light (1300 nm) combined with a coherence gate allows optically opaque materials to be studied, such as high concentration polymer solutions. Penetration depths of ~1.5 mm were possible inside the rheometer with concentrated DNA solutions (20 mg/mL). The operation of the interferometer and the associated signal analysis methods for velocity measurements have been described in previous publications^9, 15, 16^, but a detailed description of the current design is included in the SI Section 1. An improvement over our previous design is the inclusion of software demodulation (an electro-optical modulator modulates the signal at kHz frequencies to improve signal to noise ratios, which needs demodulation), which allows more convenient analysis of velocity fluctuations (SI Section 3).

The OCT apparatus is robust to sample opacity at high polymer concentrations, unlike standard laser Doppler velocimetry experiments, which typically fail with the inclusion of small amounts of multiple scattering and are thus limited to atypical contrast matched systems^66^ (SI Sections 2, 8). The data was acquired at 10 MHz and two methods to measure velocity fluctuations were performed (SI Section 3). The width of the power spectral density of the fringe signal on the detector provided the magnitude of the short time velocity fluctuations (averaged over 4 × 10^−2^ s, Fig. 4b). The mean positions of the power spectral densities were also plotted over time and these provided a measure of the long-time velocity fluctuations (averaged over 1 s, Fig. 4a).

The OCT velocimetry technique was first calibrated by measuring the velocity profile of a suspension of silica tracer particles in distilled water with no polymers added. It was ensured that the maximum velocity measured matched the velocity of the top plate of the rheometer. This is a major advantage of rheometer based OCT, rather than OCT with a simple pipe geometry, since there is always an in-built reference velocity calibrant available which is carefully regulated by the rheometer’s control loop^15^.

Velocity profiles for each concentration of DNA solution were recorded at a range of steady shear rates by measuring the shear velocity at 20 μm intervals across the rheometer gap in the vertical direction. In order to make the velocity profiles easy to compare, each velocity profile was normalized by rescaling the distance from the lower plate (*z*) by the rheometer gap size (*z*_*g*_) and rescaling the measured velocity (v) by the velocity of the top plate (*v*_*g*_) in Figs 1 and 7.

Investigations into flow instabilities often study transient behavior with velocity profiles evolving over time. Therefore, in order to gain further insight into these transient phenomena, the interferometer was set to measure the velocity at a specific depth within the rheometer gap and the velocity measured as a function of time following initiation of the shear. This was repeated at a number of depths with 50 μm intervals, and the velocity as a function of time was plotted as a colour map (Figs 1, 2 and 8).

The signals generated by the OCT velocimetry technique allowed the fluctuations of the velocity of sheared complex fluids to be quantified in two ways; at short times (4 × 10^−2^ s) and at long times (1 s) (SI Section 3). During velocity profile measurements, the velocity was recorded a number of times at a given depth, and the average velocity was calculated. The distribution of measured velocities during this time can be used to quantify the degree of velocity fluctuations at long times (1 s) in Fig. 5a. Furthermore a histogram was made of the velocities at a point close to the lower plate (Fig. 5b) and the characteristic statistical parameters (standard deviation, skew and kurtosis) were calculated (Fig. 5b inset). The rescaled standard deviations of the velocity fluctuations using the two methods at long and short times are shown in Figs 4 and 5. The amplitude of the measured velocity fluctuations was observed to vary as a function of the DNA concentration and the applied shear rate.

In order to quantify the type of flow in more detail, the relative area (*A*_*rel*_) defined by the difference between the velocity profile as a function of depth in the rheometer and a straight line connecting its end points (a virtual linear flow) was calculated and used in the creation of a phase diagram in Fig. 3 (SI Section 6). A similar method of data analysis was performed previously with shear banding experiments with polyacrylamides^9^. In the Newtonian-like regime, *A*_*rel*_ has a very small value, whereas for stable wall slip, *A*_*rel*_ has a significant finite value with a contribution from strain localization (shear banding) at the boundaries. In time evolving wall slip, *A*_*rel*_ changes with time, and when the flow becomes unsteady (detected using the velocity fluctuations as a function of time) it is classified as turbulent. *A*_*rel*_ reduces slightly in the turbulent regime (due to a reduction in both wall slip and boundary strain localization), but still has a significant value compared with the Newtonian-like regime. The dynamic phase diagram (Fig. 3) is plotted as a function of Weissenberg number (*W*_*I*_) to facilitate comparison with other experiments. The Weissenberg number is the shear rate ($\dot{\gamma }$) rescaled by the characteristic relaxation time (τ) of the solutions (measured in linear viscoelasticity experiments, Fig. S4) i.e. ${W}_{I}=\dot{\gamma }\tau$. Non-linear bulk rheology experiments were also performed (SI Section 4).

### Sample preparation

Solutions of calf thymus DNA with a molecular weight of 50 × 10^6^ g/mol (~75 k base pairs) were prepared using a TRIS buffer containing 0.1 M NaCl. The Debye screening length (λ_D_, the length scale of the electrostatic interaction) was therefore 1 nm. Similar calf thymus DNA samples were also prepared in unbuffered solutions with no added salt. The Debye screening length in this case is fairly long ranged, defined by trace ionic impurities in the DNA solutions and the autodissociation of water (10^−6^ M), λ_D_ ~ μm-mm. The calf thymus DNA was purchased from USB Co and the non-linear rheology of similar samples has been previously studied in the literature^10, 18, 20, 34^. DNA solutions of concentration 5, 10, 15 and 20 mg/ml were prepared and allowed to equilibrate for several days. Silica tracer particles with 0.5 μm radius (Polysciences) were added at a concentration of 0.1% (w/w) in order to provide the scattering needed for the velocimetry measurements.

Using the equation, ${c}^{\ast }=\frac{3{M}_{w}}{4\pi {N}_{A}{R}_{g}^{3}}$, where M_w_ is the molecular weight, N_A_ is Avogardo’s number and the radius of gyration of a DNA chain is R_g_ = 2l_p_(aN⁄12l_p_)^v^, with a = 0.34 nm, l_p_ ~ 0.05 µm, N = 75,000 and v = 0.5, the semi-dilute overlap concentration (c*) was found to be 0.062 mg/ml^10^. All of the solutions used were therefore in the semi-dilute regime. All of the solutions are also well entangled (our M_w_ is 50 × 10^6^ g/mol), since the molecular weight threshold required for entanglement of a 5 mg/ml solution is 2.1 × 10^6^ g/mol, and 5 mg/ml is the lowest polymer concentration we studied.

The Reynold’s numbers for the highest applied shear rate (100 s^−1^) were calculated to be 8.5 × 10^−5^, and 1.2 × 10^−5^ for the 5 and 20 mg/ml concentration solutions respectively (based on bulk rheology experiments, SI Section 5), confirming that all the DNA solutions we used were in a very low Reynolds number regime and classical turbulence was not expected^1, 2^.

Sample degradation at high shear rates was tested by comparing the viscosity before and after shear. No substantial changes in the viscosity were observed, meaning that substantial degradation of the polymers was not occurring during shear, since the viscosity is known to be a sensitive function of molecular weight in all polymer solutions^24, 67^.

## Electronic supplementary material

Supplementary Information
