# Supplementary material for: Elastic turbulence in entangled semi-dilute DNA solutions measured with optical coherence tomography velocimetry
Source: Sci Rep. 2017 Apr 26;7:1186. doi: 10.1038/s41598-017-01303-4 (PMC5430809; doi:10.1038/s41598-017-01303-4)
Supplement: Supplementary file 1 — Supplementary Information [file 41598_2017_1303_MOESM1_ESM.pdf]

Supplementary Information for 'Elastic turbulence in entangled semi-dilute DNA solutions measured with optical coherence tomography velocimetry', A.V.Malm, T.A.Waigh

Section 1 – Optical coherence tomography apparatus

.

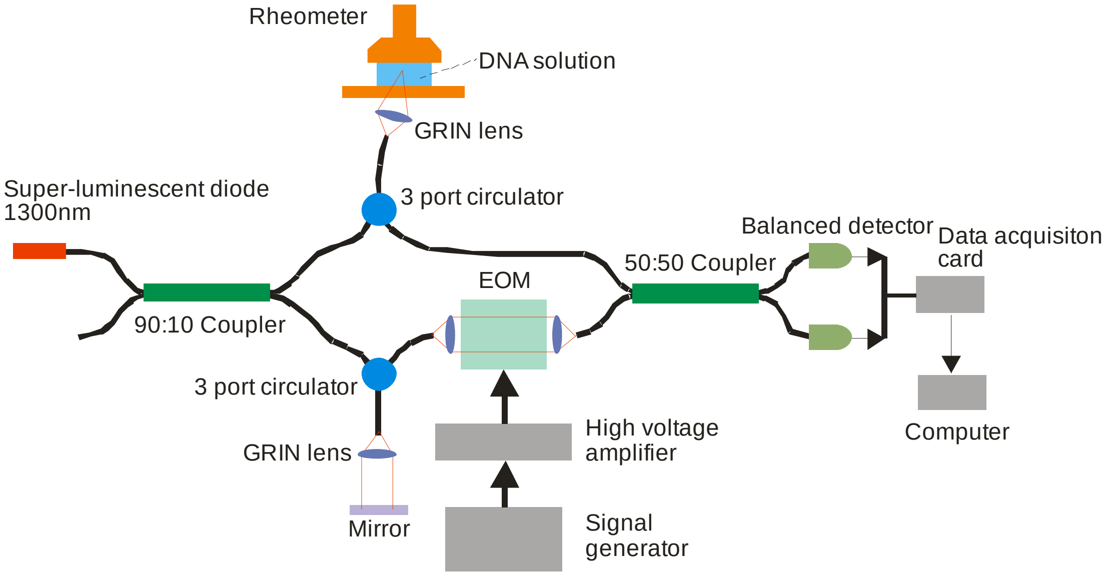

Figure S1. The optical coherence tomography velocimeter used to measure the velocity of 3.4 pL volumes of DNA solutions in a plate/plate rheometer (Bohlin Gemini). EOM is an electro optical modulator. The design involves a fibre based Mach-Zender interferometer. Infrared light is used at 1300 nm. The reference arm with the mirror is provided with a high frequency modulation using the EOM to reduce the effects of the *1/f* noise on the detector. This improves the detection of low shear rates (low velocities). A balanced detector is used to suppress common mode noise. Power spectral densities are calculated in LabView in the computer[1].

The apparatus used to measure the velocity of the DNA solutions is shown in **Figure S1**. A Mach-Zender interferometer was used at infrared wavelengths (1300 nm) to create fringes on the balanced detector. The equipment was built using fibre optics components purchased from Thorlabs e.g. couplers, grin lenses and a balanced detector. The EOM (Thorlabs EO-PM-NR-C3) was based on a Magnesium doped Lithium Nitrate crystal with a wavelength range of 1250-1650 nm. The modulation signal was applied at 20 kHz in the experiments. The light was launched through the EOM using a Thorlabs fibre port mount. Alignment of the EOM was found to be challenging without the mount. More details can be found in A.Malm et al[1,2].

Motion of samples in the rheometer caused the fringes to move and the sample's velocity (v) could be calculated from either the peak position of the power spectral density of the fringe intensity (ω) or from the phase extracted using a Hilbert Transform of the fringe intensity (which also provides a value for ω)

$$v = \frac{\lambda\omega}{2\sin\theta} \qquad \text{(S1)}$$

where θ is the angle the light made with the lower plate of the rheometer (a slight angle was needed to avoid a back reflection) and λ is the wavelength of the light used. More details can be found in M.Harvey et al[3]. The data acquisition card operated at 10 MHz i.e. fifty times faster than the Harvey et al article[1].

Software Demodulation of the Power Spectral Density (PSD) Data

To facilitate the analysis of the velocity fluctuations, demodulation of the signal from the Mach-Zender interferometer was achieved using a custom written program in LabView. Real time analysis of the velocities was based on the following algorithm. This analysis is an improvement on the method used in A.Malm et al[2], since the signal was automatically demodulated which facilitates the calculation of velocities. Firstly the signal has a high pass filter applied to it in order to remove both any low frequency 1/f noise (intrinsic to all detectors) and the unmodulated Doppler frequency peak. The signal was then multiplied by a sine wave, with a frequency equal to that of the modulation by the EOM (20 kHz). Ignoring amplitude constants, the Power (P) of the modulated signal as a function of time (*t*) can be described as

$$P(t) = \cos(\omega_D t) + \sin[(\Omega + \omega_D)t] - \sin[(\Omega - \omega_D)t] \qquad \text{(S2)}$$

where Ω is the modulation frequency and ω_D is the Doppler shift frequency due to the movements of the sample. The Power Spectrum of the signal contains peaks at the Doppler frequency, ω_D, and side-band peaks around the modulation frequency occur at Ω-ω_D and Ω+ω_D. Applying a high pass filter to this signal will remove low frequency noise and the first term in equation (S2) and multiplication by a carrier sine wave gives

$$P(t) = \sin(\Omega t)\sin[(\Omega + \omega_D)t] - \sin(\Omega t)\sin[(\Omega - \omega_D)t] \qquad \text{(S3)}$$

This can be simplified to become

$$P(t) = \sin(\omega_D t) + \frac{1}{2}\left(\sin(2\Omega + \omega_D)t - \sin(2\Omega - \omega_D)t\right) \qquad \text{(S4)}$$

The Doppler frequency component of the signal is therefore recovered at ω_D, without the presence of low frequency noise. Two additional terms that correspond to side-bands centered at twice the modulation frequency are also generated (equ S4). However these can be removed by subsequently applying a low pass filter to the signal.

Comparison of PSD versus phase analysis methods to calculate the velocity.

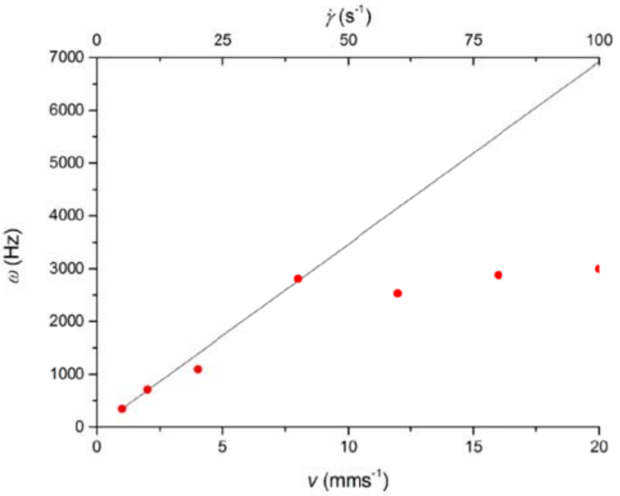

Figure S2. Comparison of the measured phase velocities (ω) by direct phase analysis with those from power spectral densities of the intensity, as a function of the shear rates $\dot{\gamma}$ (equivalent to velocities, *v*, calculated with equation S1) in the rheometer. Direct phase analysis methods function well at low velocities (red circles), but fringe hopping artefacts cause a break down at high shear rates. The solid line shows the results from a power spectral density analysis, which is much more accurate in comparison to the direct phase analysis method and was therefore predominantly used in the analysis of elastic turbulence with DNA solutions[1].

The most standard method in the OCT literature to calculate sample velocities (*v*) is phase analysis (Hilbert transforms are needed with time domain measurements)[4]. However this is found to provide erroneous values at high shear rates (Figure S2)[1]. Therefore the power spectral density method was preferred for all measurements except those at the very lowest shear rates. It is expected that many commercial OCT spectrometers will have trouble measuring fast shear rates in rheometers due to their relatively low sampling rates (we sacrificed the imaging capability for improved dynamics) and fringe hopping phenomena that are associated with phase analysis. In the literature such problems have led to a resurgence in time domain OCT methods to measure fast dynamics and hybrid frequency/time domain OCT devices have been developed[5]. In conclusion our OCT apparatus has been well optimized to provide dynamic information and we expect it is very competitive in comparison with traditional spectral domain imaging OCT devices, where fringe hopping artefacts, such as those shown in Figure S2, would limit the highest available shear rates. The fastest rate we probed (20 mm s^{-1}) is four times faster than that demonstrated by the joint spectral and time domain Doppler OCT of Szkulmowski et al[5] (2-3 times faster than Bukowska et al[6]) and this rate is limited by the inertia of the rheometer, not the signal to noise ratio of our OCT detection.

Section 2 – Detailed comparison of our apparatus with other velocimetry and OCT apparatus

Our group were the first to combine OCT detection with a rheometer and we hold a UK patent in the area[3,7]. Subsequent to our first publication a second group has combined commercial OCT apparatus with a rheometer[8] and a third group has recently performed particle tracking microrheology without a rheometer[9] (similar to a previous non-imaging study by our group on OCT microrheology, that used a correlator and was 10^8 times faster[10]).

Numerous previous studies have examined Doppler OCT with viscoelastic fluids[11-15]. However they were not performed in a rheometer, which provides a control loop to carefully control the stress and strain experienced by a sample as a function of time. Without a rheometer it is hard to accurately quantify the viscoelasticity of materials. Therefore we provide a comparison of different velocimetry techniques that have been performed in a rheometer in **Table 1**[16], where our OCT rheometer is seen to be very competitive.

**Technique**	**Spatial resolution**	**Temporal resolution**	**System requirement**	**Advantages**	**Disadvantages**
OCT shear rheometer (current design)	9 μm slices, 3.4 pL volume	10^{-7} s-1 s dynamics, ~1 s per point	Source of speckle.	High spatial and temporal resolution. Opaque materials and reasonably cheap.	1D imaging.
Standard photon correlation spectroscopy (PCS) velocimetry	100 μm slices	1 s per point	Weak scattering, no turbidity.	Reasonable spatial and temporal resolution.	No opaque materials. No imaging.
Ultrasonic velocimetry (UV)	40 μm slices	2×10^{-3}-2 s per point	Acoustic contrast.	Reasonable spatial and temporal resolution. Opaque materials.	Can require seeding
NMR	50-100 μm slices	1 s per profile	Correct nuclei.	Opaque materials	Very expensive, low resolution
Particle tracking velocimetry	1-10 μm slices	2.5×10^{-3}-1 s per profile	Tracer particles and optically transparent.	Excellent spatial and temporal resolution.	Requires seeding and transparent systems. Small depth of field.

Table 1. Comparison of velocimetry techniques that can be used with a rheometer (adapted from Manneville[16]).

More details on the apparatus can be found in the PhD thesis of Alex Malm[1], which is available online (www.escholar.manchester.ac.uk/uk-ac-man-scw:297727) and also contains a detailed analysis of the commercial advantages of the equipment.

Section 3 - Measurement of velocity fluctuations

The bulk rheometer has been carefully calibrated to provide the correct viscosity of Newtonian calibrants, such as water and glycerol. Velocity fluctuations were calculated using two different methods and both were facilitated by the automatic software demodulation of the signals. The most useful was found to be the position of the peak in the PSD signal at long times. The data was separated into 1 s chunks of time and each 1 s chunk had a power spectral density calculated. The peak in the PSD then provided a value for the velocity using equation (S1). These velocities were then histogrammed (figure 5b) and an additional PSD was taken of the time sequence of the velocities, to calculate their spectrum (as opposed to the initial PSDs necessary to calculate the velocities in the first place, calculated from the intensities on the detector).

An additional measure of the velocity fluctuations was possible at short times (4×10^{-2} s) using the width of the peak in the PSD of the fringe intensities (results shown in figure 4b). The use of the EOM allowed us to avoid anomalous broadening of the widths at low shear rates (a possible artefact previously reported in A.V.Malm et al[1,17], but now removed).

A wide variety of parameters could affect the distribution of velocities measured in a 3.4 pL volume in the rheometer and many of them are partially ignored in commercial rheometers, that tend to be optimized around the averaged stress/strain curves of standard calibrants. However, we have carefully considered the separate factors affecting the velocity fluctuations. These are:

a) The control loop of the rheometer is important, which regulates the stress and the strain experienced by a sample. This control loop causes the peak position to fluctuate considerably in Newtonian flows if the time constant of the feedback in the loop is improperly set. Indeed initial measurements (published in M.Harvey et al[3]) indicated the velocimetry measurements using OCT were more sensitive than the control loop transducers of the rheometer in some situations. Care was therefore taken to choose a fast time constant for the feedback loop in the rheometer that removed the intrinsic fluctuations. Most tellingly, such control loop fluctuations tended to have a

periodicity that was proportional to the rotation time of the upper plate, and were avoided in the data sets presented in the article.

b) The boundary effects due to the flow geometry can be significant. These effects are well defined in viscosity measurements with Newtonian calibrants. However, instabilities are known to be sensitively affected by flow geometries and they must be considered simultaneously with the type of complex fluid probed. Boundary effects are due to the nature of the phenomenon and are not an artefact in the measurements.

c) The size of the volume of the complex fluids sampled (3.4 pL in the current experiments) could affect the magnitude of the velocity fluctuations, since they are averaged over the volume. Clearly the velocity fluctuation spectrum could also depend on the sample volume (not originally addressed in the original Groisman/Steinberg Nature paper[18]) and the spectrum has a spatial scale associated with it (9 μm transverse to the flow direction).

d) Errors due to the finite resolution of the velocimeter. We have spent considerable time optimizing the resolution of the OCT apparatus (SI Section 1). The velocity fluctuation data measured are expected to be well within the capabilities of our apparatus[1].

e) Something intrinsic due to the dynamics of the complex fluids e.g. elastic turbulence. All physical measurements typically tend to involve Gaussian distributed random variables, due to the central limit theorem and the finite levels of injected noise (at the smallest length scales these are due to thermal effects). However, the high concentration DNA samples clearly have fat-tailed distributions (Fig. 5) that mark the transition to elastic turbulence. Such fat-tailed distributions cannot be explained by a)-d).

In conclusion we are confident that our velocity spectra are free of instrument noise and are intrinsic to the elastic turbulence phenomena investigated.

Section 4 - Bulk Rheology

Shear ramp experiments in the rheometer show that all of the DNA concentrations studied in a Tris buffer shear thinned (a common behavior for many solution state polymers), with a sudden change in gradient of the stress curve at round 1 s^{-1} (**figure S3a**). Thus, although the DNA samples at lower concentrations have constant Newtonian-like velocity gradient profiles across the gap (**figure 1a**), they are only reflecting one aspect of an intrinsically non-Newtonian (and non-linear) phenomenon i.e. shear thinning. Measurements of the shear stress over time for a constant shear rate show stress overshoots (**figure S3b**), which are typical of entangled polymeric solutions, with the short time dynamics controlled by the time scale for molecular relaxation e.g. the reptation time of the chain[19]. Additional bulk linear rheology experiments were

performed to measure the solution relaxation times (τ) needed to calculate the Weissenberg number (**figure 3**) and they are shown in **Figure S4** ($Wi = \tau \dot{\gamma}$). Bulk non-linear rheology measurements were also performed on DNA samples without any salt (**figure S5**). These low salt DNA samples were also observed to shear thin. These results are in good agreement with previous studies on the non-linear rheology of DNA[19-21], particularly those of the S.Q.Wang group that were performed with identical specimens.

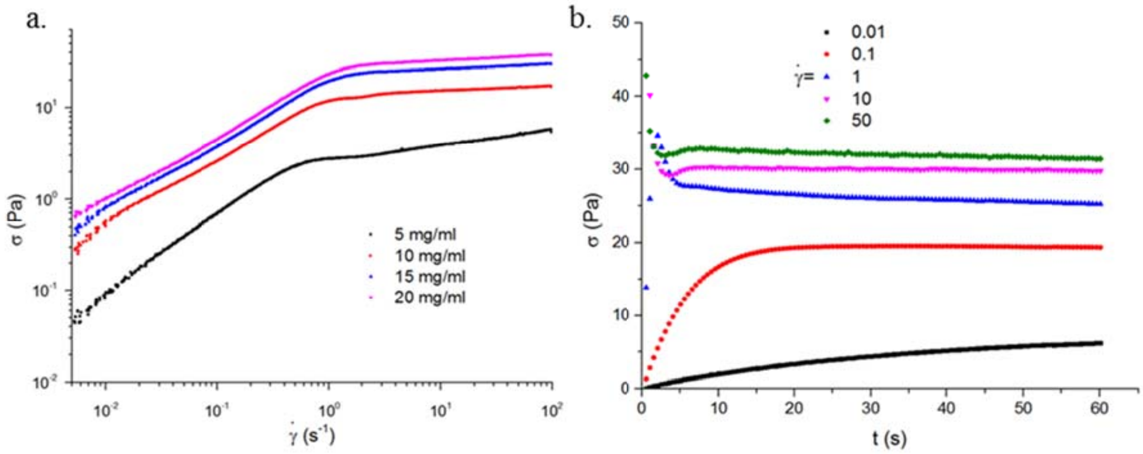

Figure S3: a) Shear stress (σ) as a function of shear rate ($\dot{\gamma}$) for four DNA concentrations (5, 10, 15 and 20 mg/mL) in a Tris buffer (high salt) measured in the Bohlin rheometer. All the solutions demonstrate shear thinning. b) The shear stress (σ) as a function of time (*t*) for a 15 mg/ml solution of DNA during shear start-up at a range of different steady shear rates (0.01, 0.1, 1, 10, 50 s^{-1}). A stress over-shoot is observed at short times (~2 s) for the higher shear rates ($\dot{\gamma} \geq 1 s^{-1}$).

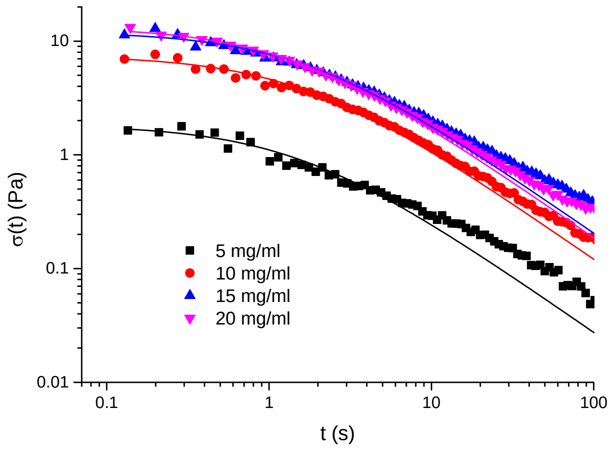

Figure S4. Stress (σ) as a function of time (t) for stress relaxation experiments in high salt DNA solutions measured using the Bohlin rheometer. Exponential relaxation functions were fit to the data (continuous) lines and used to calculate the Weissenberg numbers shown in **figure 3**. Four different DNA concentrations are shown (5, 10, 15, 20 mg/mL).

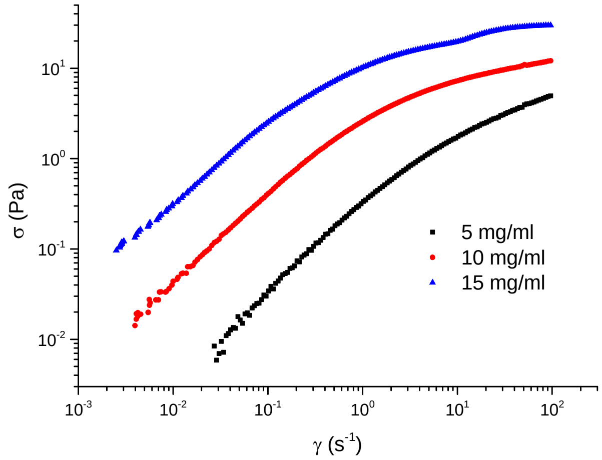

Figure S5. Stress (σ) as a function of shear rate ($\dot{\gamma}$) for stress ramp experiments in DNA solutions with no salt or buffer. Three different DNA concentrations are shown (5, 10, 15, mg/mL).

Section 5 - Power spectral density of the velocity fluctuations of silica beads in water.

As a control, the power spectral density of the velocity fluctuation of silica beads in water at high Reynolds number was measured using the OCT device (**figure S6**). The power law decay of the PSD of the velocity fluctuations ($P(\omega) \sim \omega^{-\alpha}$) has an exponent α of 1.1±0.1. This is reasonably close to that expected for Kolmogorov scaling (1.66) for classical isotropic turbulence and it is much smaller than the values measured for elastic turbulence e.g. **figure 6**. The difference between α and 1.66 is probably due to a breakdown in the assumption of homogeneous isotropic turbulence in the plate/plate geometry[22].

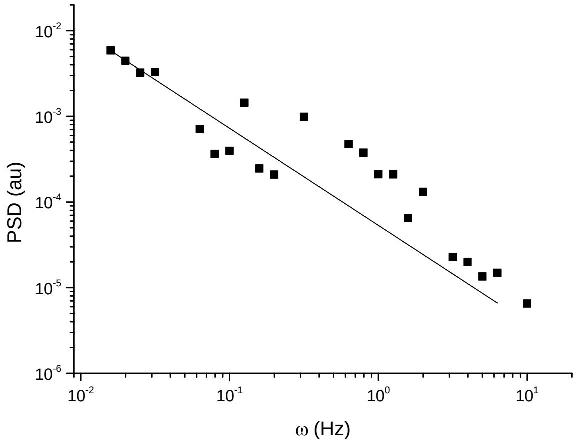

Figure S6. Power Spectral Density of the velocity fluctuations of a suspension of silica tracer particles in water (no DNA is added) sheared in the rheometer at 100 s^{-1}. A single power law dependence ($P(\omega) \sim \omega^{-\alpha}$) is observed with α = 1.1±0.1.

Section 6 – Analysis of velocity profiles using A_{rel}

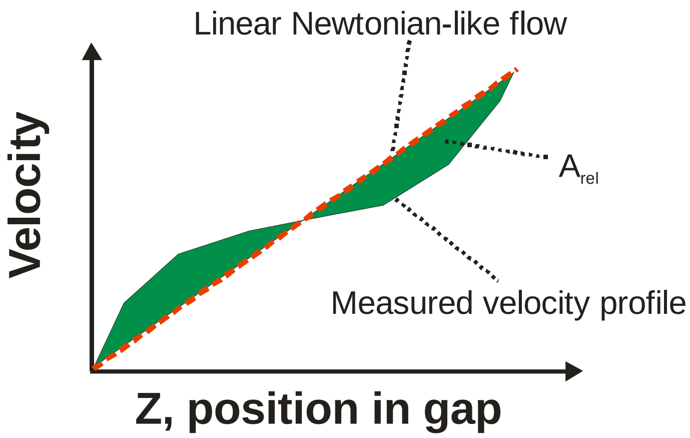

Figure S7. Schematic diagram that demonstrates the calculation of the area parameter (A_{rel}, green) used in **figure 3**. A_{rel} is the area between the measured velocity profile as a function of the vertical position in the gap of the parallel plate rheometer and a virtual linear Newtonian-like flow profile (red).

A_{rel} is introduced as a new method to classify the type of non-linear flow patterns[23]. It combines a measure of both wall slip and non-linear velocity profiles (i.e. shear banding) as a function of gap position. Previous methods in the literature have tried[24] to define a slip length following previous work of de Gennes. However such slip lengths tend to sensitively depend on the details of the extrapolation procedure. They work fine for linear velocity gradients, but for non-linear flow profiles such as those found with DNA, it is hard to quantify the slip length with any degree of accuracy. We therefore use A_{rel}, instead of the slip length, as it is a more robust parameter. Interestingly, A_{rel} decreases in the elastic turbulence regime compared with the wall slip regime, which indicates the close relationship between the two phenomena i.e. elastic turbulence suppresses wall slip and boundary strain localization in this case.

Section 7 – Elastic Instabilities

Non-linear instabilities, such as elastic turbulence, have also been called rheological chaos in the literature[25-27]. However we believe that chaos encompasses too wide a range of non-linear instabilities and as such is too generic as a description. Elastic turbulence is a much more specific succinct descriptor of the experimental phenomena observed, since the Reynold's number can be swapped for the Weissenberg number to provide a series of useful analogies. Other Low Reynolds number flow instabilities also now use the analogy to classical turbulence, such as bacterial turbulence[28] and active turbulence[29].

Lots of seminal work has been performed on elastic turbulence by the Steinberg group. However we believe there are some misconceptions in their articles. In Liu et al[30] neutral polymer theories are used to analyse the dynamics of charged polymers. This led the authors to develop a new empirical relationship for their

relaxation times, which was very similar to the results of Dobrynin et al[31], but without a solid underlying physical understanding. This led Liu et al to overestimate the semi-dilute concentration in their specimens and many of the solutions in which they have studied elastic turbulence may have been in the semi-dilute regime[30].

Therefore elastic turbulence appears to commonly occur in semi-dilute polymeric solutions, although the related phenomenon of elasto-inertial turbulence is thought to occur at high shear rates in dilute solutions[32] and has been repeated in subsequent studies[33].

Section 8 - Comparison of OCT velocimetry with laser Doppler velocimetry (LDV) and particle imaging velocimetry (PIV) used previously to examine elastic turbulence in low concentration polymer solutions.

LDV was used in the initial studies of elastic turbulence[18]. The technique is restricted to transparent samples[34] and the inclusion of small amounts of multiple scattering invalidates the process of analysis. Furthermore fringe contrast is lost at high sample concentrations and necessitates contrast matching of samples. LDV could thus not be used with the high concentration DNA samples described in the current article. LDV requires two intersecting beams which makes alignment more challenging and the observation volume sensitively depends on the intersection angle (it is not constant across the gap). For comparison OCT has a single beam, is confocal and thus has a much smaller uniform interaction volume and better background rejection.

PIV is also restricted in terms of sample opacity when compared with OCT, has lower detection rates (limited by the camera speed) and requires much slower, more intensive data analysis procedures (thus it is more challenging to measure the velocity fluctuations).

References

1 Malm, A. V. *Optical coherence tomography velocimetry and X-ray Scattering Rheology of Complex Fluids*, University of Manchester, (2015).

2 Malm, A. V., Harrison, A. W. & Waigh, T. A. Optical coherence tomography velocimetry of colloidal suspensions. *Soft Matter* **10**, 8210-8215 (2014).

3 Harvey, M. & Waigh, T. A. Optical coherence tomography velocimetry in controlled shear flow. *Physical Review E* **83**, 31502 (2010).

4 Fercher, A. F., Drexler, W., Hitzenberger, C. K. & Lasser, T. Optical coherence tomography - principles and applications. *Reports on Progress in Physics* **66**, 239-303 (2003).

5 Szkulmowski, M., Szkulmowska, A., Bajraszewski, T., Kowalczyk, A. & Wojtkowski, M. Flow velocity estimation using joint spectral and time domain optical coherence tomography. *Optics Express* **16**, 6008-6025 (2008).

6 Bukowska, D. M. *et al.* Assessment of the flow velocity of blood cells in a microfluidic device using joint spectral and time doman optical coherence tomography. *Optics Express* **21**, 24025-24038 (2013).

7 Waigh, T. A. & Harvey, M. High resolution optical coherence tomography rheometry. UK patent, M.Harvey, T.A.Waigh, High resolution optical coherence tomography rheometry patent (2010).

8 Haavisto, S., Koponen, A. I. & Salmela, J. New insight into rheology and flow properties of complex fluids with Doppler optical coherence tomography. *Frontiers in Chemistry* **2**, 27 (2014).
9 Chu, K. K. *et al.* Particle-tracking microrheology using micro-optical coherence tomography. *Biophysical Journal* **111**, 1053-1063 (2016).
10 Sharma, R. C., Papagiannopoulos, A. & Waigh, T. A. Optical coherence tomography picorheology of biopolymer solutions. *Applied Physics Letters* **92**, 173903 (2008).
11 Wang, X. J., Milner, T. E. & Nelson, J. S. Characterization of fluid-flow by optical doppler tomography. *Optics Letters* **20**, 1337-1339 (1995).
12 Wang, X. J., Milner, T. E., Chen, Z. P. & Nelson, J. S. Measurement of fluid-flow-velocity profile in turbid media by the use of optical Doppler tomography. *Applied Optics* **36**, 144-149 (1997).
13 Chen, Z. P., Milner, T. E., Dave, D. & Nelson, J. S. Optical Doppler tomographic imaging of fluid flow velocity in highly scattering media *Optics Letters* **22**, 64-66 (1997).
14 Izatt, J. A., Kulkami, M. D., Yazdanfar, S., Barton, J. K. & Welch, A. J. In vivo bidirectional color Doppler flow imaging of picoliter blood volumes using optical coherence tomography. *Optics Letters* **22**, 1439-1441 (1997).
15 White, B. R. *et al.* In vivo dynamic human retinal blood flow imaging using ultra-high speed spectral domain optical Doppler tomography. *Optics Express* **11**, 3490-3497 (2003).
16 Manneville, S. Recent experimental probes of shear banding. *Rheol. Acta* **47**, 301-318 (2008).
17 Malm, A. V., Waigh, T. A., Jaradat, S. & Tomlin, R. Optical coherence tomography velocimetry with complex fluids. *Journal of Physics: Conference Series* **602**, 12039 (2015).
18 Groisman, A. & Steinberg, V. Elastic turbulence in a polymer solution flow. *Nature* **405**, 53 (2000).
19 Boukany, P. E. & Wang, S. Q. Shear banding or not in entangled DNA solutions depending on the level of entanglement. *Journal of Rheology* **53**, 73 (2009).
20 Boukany, P. E. & Wang, S. Q. Shear banding or not in entangled DNA solutions. *Macromolecules* **43**, 6950-6952 (2010).
21 Boukany, P. E. & Wang, P. E. Exploring the transition from wall slip to bulk shear banding in well entangled DNA solutions. *Soft Matter* **5**, 780-789 (2009).
22 Frisch, U. *Turbulence: The legacy of A.N.Kolmogorov*. (CUP, 1995).
23 Jaradat, S., Harvey, M. & Waigh, T. A. Shear-banding in polyacrylamide solutions revealed via optical coherence tomography velocimetry. *Soft Matter* **8**, 11677 (2012).
24 Wang, S. Q., Ravindranath, S. & Boukany, P. E. Homogenous shear, wall slip and shear banding of entangled polymeric liquids in simple-shear rheometry: a roadmap of nonlinear rheology. *Macromolecules* **44**, 183-190 (2011).
25 Cates, M. E., Head, D. A. & Ajdari, A. Rheological chaos in a scalar shear-thickening model. *Physical Review E* **66**, 25202 (2002).
26 Ganapathy, R. & Sood, A. K. Intermittency route to rheochaos in wormlike micelles with flow-concentration coupling. *Physical Review Letters* **96**, 108301 (2006).
27 Cates, M. E. & Fielding, S. M. Rheology of giant micelles. *Advances in Physics* **55**, 799-879 (2006).
28 Dunkel, J. *et al.* Fluid dynamics of bacterial turbulence. *Physical Review Letters* **110**, 228102 (2013).
29 Hemingway, E. J. *et al.* Active viscoelastic matter: from bacterial drag reduction to turbulent solids. *Physical Review Letters* **114**, 98302 (2015).
30 Liu, Y., Jun, Y. & Steinberg, V. Concentration dependence of the longest relaxation time of dilute and semi-dilute polymer solutions. *Journal of Rheology* **53**, 1069-1085 (2009).
31 Dobrynin, A. V., Colby, R. H. & Rubinstein, M. Scaling theory of polyelectrolyte solutions. *Macromolecules* **28**, 1859-1871 (1995).
32 Samanta, D. *et al.* Elasto-inertial turbulence. *PNAS* **110**, 10557-10562 (2013).
33 Vonlanthen, R. & Monkewitz, P. A. *Journal of Fluid Mechanics* **730**, 76-98 (2013).
34 Shapley, N. C., Armstrong, R. C. & Brown, R. A. Laser doppler velocimetry measurements of particle velocity fluctuations in a concentrated suspension. *Journal of Rheology* **46**, 241-271 (2002).
